# Anterior Segment Imaging in Ocular Surface Squamous Neoplasia

**DOI:** 10.1155/2016/5435092

**Published:** 2016-10-05

**Authors:** Sally S. Ong, Gargi K. Vora, Preeya K. Gupta

**Affiliations:** Duke University Department of Ophthalmology, Durham, NC, USA

## Abstract

Recent advances in anterior segment imaging have transformed the way ocular surface squamous neoplasia (OSSN) is diagnosed and monitored. Ultrasound biomicroscopy (UBM) has been reported to be useful primarily in the assessment of intraocular invasion and metastasis.* In vivo* confocal microscopy (IVCM) shows enlarged and irregular nuclei with hyperreflective cells in OSSN lesions and this has been found to correlate with histopathology findings. Anterior segment optical coherence tomography (AS-OCT) demonstrates thickened hyperreflective epithelium with an abrupt transition between abnormal and normal epithelium in OSSN lesions and this has also been shown to mimic histopathology findings. Although there are limitations to each of these imaging modalities, they can be useful adjunctive tools in the diagnosis of OSSN and could greatly assist the clinician in the management of OSSN patients. Nevertheless, anterior segment imaging has not replaced histopathology's role as the gold standard in confirming diagnosis.

## 1. Introduction

Ocular surface squamous neoplasia (OSSN) is the most common tumor affecting the ocular surface in adults [1]. OSSN was a term suggested by Lee and Hirst to describe all primary dysplastic and carcinomatous lesions that originate from the epithelium of the cornea or conjunctiva [2]. Histologically, OSSN includes epithelial dysplasia, carcinoma in situ, and invasive squamous cell carcinoma [3]. In conjunctival and corneal intraepithelial neoplasia (CCIN), epithelial cells are thickened, dysplastic, and irregular with increased cell proliferation. These changes affect less than the full thickness of the epithelium. When the entire epithelium is involved but tumor cells have not yet invaded the substantia propria, the lesion is categorized as carcinoma* in situ*. Invasive squamous cell carcinoma is defined as when the lesion has affected the epithelial basement membrane and substantia propria [4, 5]. It can locally invade the sclera, uvea, eyelids, and orbit and has the ability to metastasize to distant sites thus potentially becoming life threatening [6].

**Figure 1:**
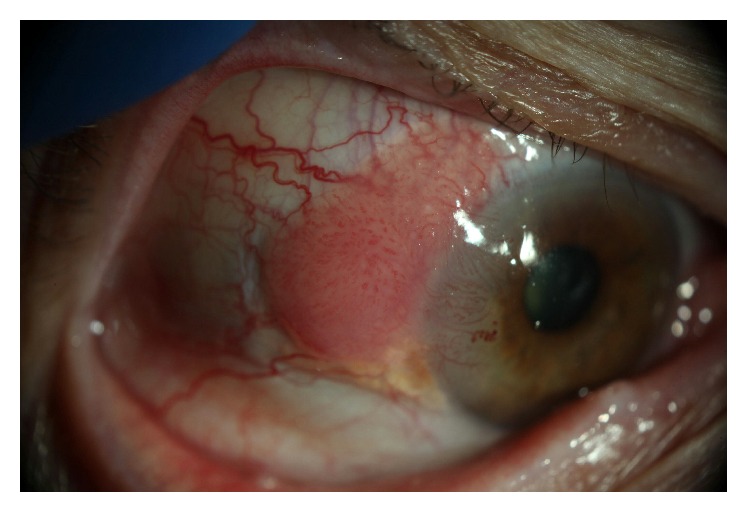
Slit lamp photograph of a corneal-conjunctival intraepithelial neoplasia with gelatinous and papilliform features as well as feeder vessels.

OSSN occurs worldwide but has the highest incidence rates in Africa [7]. Risk factors for developing OSSN include solar ultraviolet radiation as well as human immunodeficiency virus (HIV) and human papillomavirus (HPV) infections [7, 8]. OSSN lesions are usually located within the interpalpebral fissure at the limbus in the nasal quadrant, which receives the highest intensity of sunlight [7]. Clinically, OSSN has been described as elevated gelatinous, papilliform, or leukoplakic limbal lesions that move freely over the sclera with adjacent feeder vessels (Figure 1) [4, 9]. Diagnosis can be made by clinical examination with slit lamp biomicroscopy. However, overlap in clinical features in OSSN and masqueraders like pterygium, dyskeratosis, papilloma, scar tissue, corneal pannus, pyogenic granuloma, amelanotic melanoma, and sebaceous cell carcinoma can occasionally make diagnosis by clinical examination alone difficult. Accuracy of clinical diagnosis has been reported to range between 40% and 86% when compared to histopathology results [2, 10].

The gold standard for confirming diagnosis of OSSN is excisional biopsy for histopathology. This technique, however, is not without its limitations. Biopsy for histopathology may miss lesions that are not included in the excised tissue as diffuse lesions can be difficult to excise with clear margins. Additionally, since OSSN can recur even after successful treatment, repeated excisional biopsies may cause conjunctival scarring and limbal stem cell deficiency [13]. Adjunctive methods such as impression cytology (IC) and vital dye staining have therefore been used to assist in the diagnosis and follow-up of OSSN.

Although now rarely done, IC can be useful in diagnosis and has been shown to correlate closely with histopathology [14, 15]. In IC, superficial epithelial cells are collected by applying collecting devices (either cellulose acetate filter papers or Biopore membrane device [Millipore Corp, Bedford, MA]) such that the cells adhere to the surface and are removed from the eye to be fixed, stained, and then mounted on a slide for analysis [29]. Nolan et al. found that 55% of intraepithelial OSSN cases diagnosed by IC had keratinized dysplastic cells often accompanied by hyperkeratosis, 35% had large syncytial-like groups, and 10% had nonkeratinized dysplastic cells as a predominant feature [16]. Importantly, however, it was not possible to differentiate intraepithelial lesions from invasive squamous cell carcinoma given the superficial sampling of cells, thus limiting the utility of IC in diagnosing invasive disease [16]. The inability of IC to reach deep atypical cells even with repeated imprints of the same area of the lesion has also been noted in other studies [17, 18].

Another diagnostic test that is inexpensive and helpful in identifying OSSN is dye staining. Diagnostic dyes like lissamine green and rose bengal are routinely used to stain and delineate the extent of OSSN lesions but since these dyes are nonspecific and stain many other ocular surface conditions, it is not possible to diagnose OSSN with the use of these dyes alone. Other vital dyes that have been studied in the diagnosis of OSSN include toluidine blue (ToB) and methylene blue. ToB and methylene blue are acidophilic dyes that stain abnormal tissue dark royal blue. They have an affinity for nucleic acids and, given the increased nuclear material from high rates of mitoses and poor cell-to-cell adhesion in malignancy, these tissues stain more frequently than benign tissues [22]. Several studies have shown that ToB and methylene blue staining have a high sensitivity but low to moderate specificity in diagnosing OSSN compared to histopathology [20–22]. This makes ToB and methylene blue a good initial screening tool since very few OSSN lesions did not stain with these dyes but an insufficient diagnostic modality since a high proportion of benign lesions also stained positive [22].

Given the limitations of IC and vital dye staining, there is now increased interest in the use of anterior segment imaging techniques to assist in the diagnosis of OSSN. This is becoming especially pertinent since current management options for OSSN include not only surgical excision with cryotherapy but also primary medical therapy with the use of topical chemotherapy such as mitomycin-C, 5-fluorouracil, and interferon alfa-2b. In this review, we will discuss the use of ultrasound biomicroscopy (UBM),* in vivo* confocal microscopy (IVCM), and anterior segment optical coherence tomography (AS-OCT) in the diagnosis and monitoring of OSSN. We performed a comprehensive review within the peer reviewed literature using http://pubmed.gov/. The following search terms were used: ocular surface squamous neoplasia, conjunctival intraepithelial neoplasia, corneal intraepithelial neoplasia, carcinoma* in situ* squamous cell carcinoma, impression cytology, toluidine blue, methylene blue, ultrasound biomicroscopy,* in vivo* confocal microscopy, and anterior segment optical coherence tomography.

## 2. Ultrasound Biomicroscopy

Ultrasound biomicroscopy (UBM), which was first developed by Pavlin et al. in 1990, provides cross-sectional visualization of the anterior segment in an intact globe at microscopic resolution [30]. UBM uses high frequency ultrasonography ranging from 20 to 50 MHz. In the 50 MHz mode, images to a depth of 5 to 6 mm at a resolution of 25 microns can be produced [31]. Pavlin et al. suggested the use of UBM to measure and determine the extent of invasion of anterior segment tumors, which had been difficult with conventional ultrasound [32, 33]. Today it is widely used to image anterior segment tumors although limitations exist. These include requiring an eyebath in the reclined position and a technician familiar with its use to obtain the best images.

Studies on the use of UBM in diagnosing OSSN have shown that UBM is most useful in assessing intraocular tumor extension and metastasis [11, 23]. Char et al. examined four patients with possibly highly invasive squamous cell carcinoma of the conjunctiva who underwent 20 MHz high frequency ultrasound [23]. In all four cases, UBM was useful as an adjunct to clinical examination in determining the presence of deep invasion. For example, one patient was referred for possible deep invasion from a conjunctival squamous cell carcinoma. There was no evidence for invasion on high frequency ultrasound, which correlated with biopsy findings of tumor confined to the conjunctiva. Another patient had atypical scleritis with a large superficial tumor and clinical evidence of intraocular invasion, which was confirmed by high frequency UBM showing invasion into the ciliary body with thickening [23].

**Figure 2:**
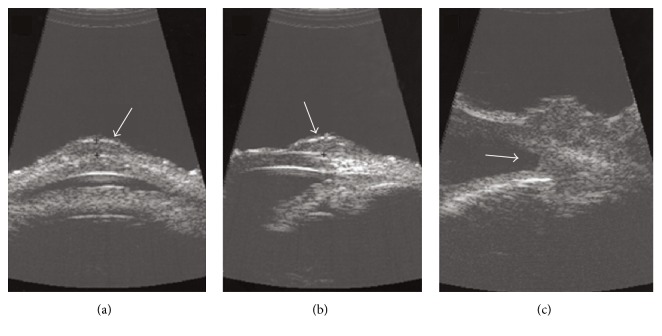
20 MHz transverse (a) and longitudinal (b) ultrasound biomicroscopy sections of conjunctival intraepithelial neoplasia demonstrate hyperechoic tumor surface (arrows) and hypoechoic stroma. (c) 20 MHz UBM image taken from a patient with squamous cell carcinoma demonstrates blunting of the anterior chamber angle (arrow) which correlated to anterior chamber angle invasion on histopathology [11].

Finger et al. described general ultrasonographic characteristics of conjunctival intraepithelial neoplasia and squamous cell carcinoma in addition to UBM findings in intraocular tumor extension in 11 patients [11]. Using 20 and 50 MHz high frequency ultrasound, the tumor surface was found to be hyperechoic while the tumor stroma was generally hypoechoic in all patients (Figures 2(a) and 2(b)). The authors also reported two UBM findings suggestive of ocular tumor extension: (1) blunting of the anterior chamber angle (Figure 2(c)) and (2) uveal thickening, which correlated with histopathology findings. In patients where the tumor had covered a functioning filtering bleb or obscured the corneal surface, the authors were able to determine that there was no evidence of intraocular extension by using UBM. In patients with orbital extension, the authors differentiated the relatively hypoechoic tumor from the more hyperechoic orbital tissues using UBM. However, imaging of the posterior margins of the tumor was limited by the maximum penetration of 20 and 50 MHz UBM. Additionally, while 50 MHz images had better resolution, 20 MHz ultrasonography provided a deeper and wider field of view. The authors concluded that UBM enabled the preoperative assessment of conjunctival tumors for intraocular invasion [11].

## 3. *In Vivo* Confocal Microscopy

*In vivo* confocal microscopy (IVCM) is a noninvasive imaging technique that allows* in vivo* microscopic examination of all layers of the ocular surface. In brief, it utilizes a point light source that scans the ocular surface and a point detector to increase the resolution [34]. Using conjugate pinholes, the point light source and the detector work in tandem to amplify the optical resolution, thus allowing the sectioning of the ocular surface at the cellular level [34]. Duchateau et al. were the first to examine conjunctival intraepithelial neoplasia using IVCM [35].

Several other reports in the literature have suggested that IVCM may be helpful in establishing the diagnosis of OSSN. Single case reports by Malandrini et al. and Wakuta et al. described IVCM findings of enlarged, irregular cells with bright hyperreflective nuclei in conjunctival and corneal intraepithelial neoplasia [36, 37]. Meanwhile, Falke et al. presented a case of carcinoma* in situ* with IVCM findings of regular conjunctival epithelium interspersed with complexes of enlarged cells with polymorphic nuclei [38].

Balestrazzi et al. described an atypical case of OSSN in a patient one month after clear corneal phacoemulsification with papillomatous invasion in the area of the side port incision. IVCM demonstrated typical characteristics of the limbal portion of OSSN with very bright intracellular bodies, while the corneal lesion demonstrated large hyperreflective round to oval shaped cells with peripherally displaced nucleus and stromal invasion of neoplasia across an interrupted Bowman layer [39]. The authors hypothesized that the Bowman layer was interrupted by the side port incision made during cataract surgery.

Gentile et al. presented a case report of how IVCM was performed to determine the involvement of corneal incisions from previous refractive surgery [40]. The patient had a history of radial keratotomy (RK) and laser* in situ* keratomileusis (LASIK), and she presented with biopsy proven limbal and conjunctival OSSN. IVCM showed that atypical cells had extended just below the level of basement membrane and Bowman layer along the scars of RK incisions. Because of these findings, the patient underwent surgical excision with a lamellar keratectomy and cryotherapy, followed by topical chemotherapy a few weeks later.

**Figure 3:**
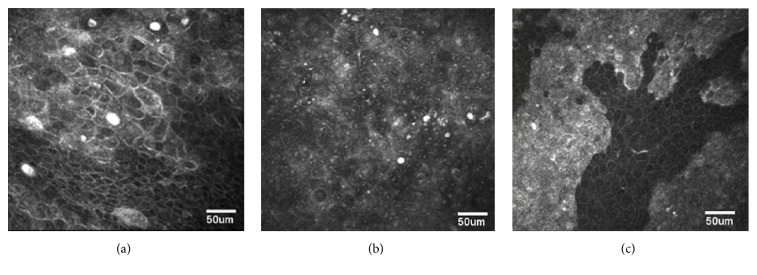
*In vivo* confocal microscopy findings of a patient with corneal/conjunctival intraepithelial neoplasia (CCIN). (a) demonstrates multinucleated bizarre-shaped cells in the mid-epithelial layer. In (b), a starry-sky pattern (ill-defined borders with tiny bright spots 2 to 4 *μ*m in size within dark spaces) is seen in the basal cells. (c) demonstrates the fimbriated advancing border of CCIN at the mid-epithelial layer. There is higher reflectivity and cell density as well as pleomorphism in CCIN compared to the adjacent normal epithelium [12].

Larger case series by Alomar et al., Parrozzani et al., and Xu et al. also demonstrated correlation between IVCM and histopathology findings [12, 24, 25]. Alomar et al. studied 4 patients with corneal/conjunctival intraepithelial neoplasia (CCIN) and reported that, in these lesions, bright prominent nucleoli produced a starry night sky pattern [12]. These lesions also consisted of hyperreflective pleomorphic cells, which resulted in a contrast between the edge of the darker normal cells and the lesions with hyperreflective cells (Figure 3). Additionally, the authors noted that subbasal corneal nerves were absent in areas of CCIN. Parrozzani et al. examined 10 cases of OSSN and reported that IVCM demonstrated dysplastic cells in each case and morphologic agreement with* ex vivo* scraping cytology and histology in 100% of cases [24]. Xu et al. examined five patients with OSSN and demonstrated high concordance between the morphological features and extent of invasion shown in IVCM and histopathologic analysis [25].

The largest study thus far on the utility of IVCM in differentiating OSSN from benign lesions was conducted by Nguena et al. in Moshi, Tanzania [10]. The study recruited 60 cases and 60 age matched controls. IVCM was attempted on all participants, and final analysis of IVCM scans was performed on 44 cases (with both histopathology and adequate scans) and 57 controls. Of the 44 cases, 18 were benign lesions and 26 were OSSN lesions as determined by histopathology. All scans were graded in a masked manner and were examined for hyperreflective cells, variation of cell size, mitotic cells, and starry night appearance of the basal layer. In each of these graded features, there was a statistically significant difference between the normal controls and cases (benign and OSSN combined) but there was no difference between the benign and OSSN cases. Therefore, this study showed that it was not possible to reliably differentiate benign from OSSN lesions because of an overlap in IVCM features in the various ocular lesions [10].

Other limitations of IVCM include its ability to provide only en face images in contrast to cross-sectional images obtained in tissue histology [12]. Additionally, it is difficult to obtain IVCM images and biopsy specimens from the exact same site where the tissue is being examined [10]. Moreover, because it provides images at a cellular level, IVCM is unable to provide a comprehensive scan of the entire ocular surface.

## 4. Anterior Segment Optical Coherence Tomography

First introduced by Izatt et al. in 1994, anterior segment optical coherence tomography (AS-OCT) is a noncontact and noninvasive imaging technique that captures high resolution cross-sectional images of the anterior segment [41]. In AS-OCT, the Michelson interferometer is used to produce a reference beam of infrared light [42, 43]. The reference beam of light is then collected along with light reflected from the tissue sample to create an interference pattern. Multiple interference patterns are created over the surface of the sample being imaged [42]. The delay of tissue reflections against the reference beam of light is compared to create a series of axial scans (A-scans), which are then combined into a composite image [44].

In the original time-domain OCT (TD-OCT), axial resolution was limited at 18 *μ*m. In a study comparing TD-OCT with UBM, Bianciotto reported that while TD-OCT was useful for the assessment of superficial nonpigmented lesions such as conjunctival tumors, UBM was in general superior for the visualization of all tumor margins and had fewer problems with posterior tumor shadowing [31]. UBM provided superior overall image quality and tumor visualization, improved resolution of the posterior margin, and much better resolution of pigmented tumors, iris pigment epithelium cysts, and ciliary body lesions [31].

With the development of spectral domain OCT (SD-OCT), higher resolution imaging has become available. High resolution OCT (HR-OCT) is capable of providing axial resolution of 5–10 *μ*m, while ultra-high resolution OCT (UHR-OCT) can provide axial resolution better than 5 *μ*m [42]. Vajzovic et al. demonstrated that a custom built UHR-OCT with axial resolution of 2 *μ*m allowed the delineation of individual cornea layers [45]. The authors also reported that UHR-OCT of an OSSN lesion showed epithelial thickening and increased reflectivity of the epithelium with an obvious delineation from tumor to nonaffected tissue [45].

**Figure 4:**
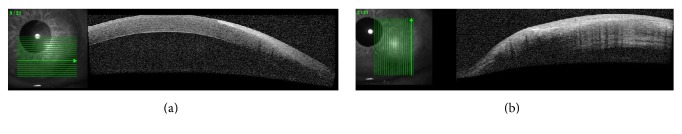
High resolution anterior segment optical coherence tomography of a corneal intraepithelial neoplasia demonstrates (a) a sharp delineation between normal and abnormal epithelium and (b) a thickened and hyperreflective epithelium.

Several subsequent studies have further demonstrated that thickened hyperreflective epithelium, abrupt transition from normal to abnormal epithelium, and a sharp plane of cleavage between the lesion and underlying tissue (Figure 4) were all features that were both seen in UHR-OCT images and histopathologic specimens of OSSN lesions [13, 26, 27]. Shousha et al. examined a case series of 7 eyes with corneal/conjunctival intraepithelial neoplasia (CCIN) and found that UHR-OCT images taken before initiation of treatment were well correlated with histopathologic specimens in the 4 cases that underwent incisional biopsies [13]. Another study by Shousha et al. of 54 eyes with biopsy proven ocular surface lesions, of which 19 were OSSN lesions, also confirmed these observations [27].

Kieval et al. compared UHR-OCT of pterygia with OSSN [26]. Pterygia have normal thin conjunctival epithelium with underlying subepithelial hyperreflective tissue. Using UHR-OCT at a resolution of 2 *μ*m, Kieval et al. showed that an epithelial thickness value greater than 140 *μ*m provided 94% sensitivity and 100% specificity for differentiating CCIN from pterygia [26]. In contrast, using HR-OCT with a resolution of 5–7 *μ*m, Nanji et al. demonstrated that an epithelial thickness cutoff at greater than 120 *μ*m provided 100% sensitivity and specificity for differentiating OSSN from pterygia [28]. In fact, normal epithelium overlying subepithelial lesion confidently rules out OSSN [27]. UHR-OCT can also be used to diagnose pigmented CCIN, as demonstrated in the study by Shousha et al., where UHR-OCT demonstrated thickened and hyperreflective epithelium in a pigmented conjunctival lesion that had been referred for conjunctival melanoma. Histopathology confirmed the diagnosis of pigmented CCIN [27].

UHR-OCT can also be used to monitor disease resolution and detect residual subclinical disease. For lesions treated successfully with topical agents, posttreatment UHR-OCT showed normalization of epithelial architecture at the site of the treated lesions. However, in lesions resistant to medical treatment, UHR-OCT will show persistently thickened epithelium with retained abrupt transition between normal and diseased epithelium [13, 42]. Continuation of topical treatment in patients with residual subclinical disease in the study by Shousha et al. resulted in complete resolution of the otherwise subclinical lesion [13]. Therefore, UHR-OCT prevented what could have been premature cessation of topical treatment, which could have increased the risk of recurrent disease. Thomas et al. reported that, in their early experience of these cases, there was a median delay between clinical and UHR-OCT resolution of approximately 16 weeks, with the longest delay being approximately 29 weeks [42]. The authors therefore suggested continuing treatment for 16 weeks after clinical resolution of disease if UHR-OCT was not available to monitor for presence of subclinical disease.

Other scenarios where UHR-OCT can be useful include ruling out OSSN in the setting of complex ocular pathology and in clinically indeterminate lesions. Thomas et al. described a patient with a past medical history of HIV, vernal keratoconjunctivitis, limbal stem cell deficiency (LSCD), and previously treated OSSN who presented with a change in appearance in the limbal conjunctiva [42]. UHR-OCT imaging revealed epithelial thickening and hyperreflectivity. After the patient completed treatment, UHR-OCT was also used to confirm resolution. UHR-OCT has also been used to show foci of OSSN in pterygia, Salzmann's nodular degeneration, HSV keratopathy, and atypical peripheral corneal infiltrates when the clinical diagnosis was unclear [42].

Additional advantages of OCT over other forms of anterior segment imaging include its noncontact method of obtaining images, patients being imaged sitting in an upright and comfortable position, and user friendliness for the operator [31]. However, due to the cost of the machine, high resolution OCT may not be readily available in resource poor settings, thus limiting its widespread use.

## 5. Conclusion

There are several adjunctive diagnostic modalities available that can assist in the diagnosis and monitoring of OSSN lesions. These include IC, vital dye staining, ultrasound biomicroscopy, IVCM, and AS-OCT. A summary of main findings from major studies on these diagnostic modalities is presented in Table 1 and a summary of advantages and disadvantages of each diagnostic modality is shown in Table 2.

Given the limitations of IC and vital dye staining, there has been a shift in interest to anterior segment imaging modalities such as UBM, IVCM, and AS-OCT. As discussed in this review, anterior segment imaging can provide the clinician with microscopic lesion detail to make an accurate diagnosis but is equally as important to guide therapeutic decisions. Each device has its limitations, but when combined with clinical examination, anterior segment imaging can greatly aid the clinician. Nevertheless, it is important to note that none of these imaging modalities has replaced histopathology's role as the gold standard for diagnosing OSSN. More research over time and advances in technology will likely provide us with further improved imaging modalities, but to date these devices warrant integration into clinical practice.

## Figures and Tables

**Table:** (a)

Impression cytology (IC)					
Study	Location	Year	Study design	Number of eyes	Main findings
Nolan et al. [14]	Australia	1994	Observational	Invasive squamous cell carcinoma (6) Conjunctival intraepithelial neoplasia (49) No OSSN (21)	IC can be used to demonstrate the morphology of normal and abnormal conjunctival cells. Cytology report was positive in 77% of histopathology reports in the moderate dysplasia to microinvasive carcinoma group. There were no false positives.

Tole et al. [15]	Australia	2001	Prospective case series	Squamous cell carcinoma (SCC) (1) Carcinoma *in situ* (2) Keratinizing dysplasia (15) Nonkeratinizing dysplasia (7)	IC is accurate in predicting the diagnosis of OSSN. Correlation rate of IC with histological findings was accurate in 80% of cases, poor in 12% of cases, and not correlated in 8% of cases. There were no false positives.

Nolan et al. [16]	Australia	2001	Retrospective observational	Intraepithelial OSSN or corneal/conjunctival intraepithelial neoplasia (142) Invasive OSSN or SCC (23)	The cytomorphology of OSSN is described in detail. For intraepithelial lesions, these include (1) keratinized dysplastic cells often accompanied by hyperkeratosis (55%), (2) syncytial-like groupings (35%), and (3) nonkeratinized dysplastic (10%) cells. Meanwhile, invasive cases had a tendency to be more highly keratinized and to have a greater degree of inflammation than the keratinized high grade intraepithelial cases but it was not possible to confidently predict invasion on IC. Sensitivity of IC in diagnosing OSSN was 78% overall but was lower (70%) when the lesion was invasive by histopathology.

Tananuvat et al. [17]	Thailand	2008	Retrospective case series	OSSN (50) including SCC (20), dysplasia (20), squamous papilloma (4), and nondysplastic changes of the epithelia (6) Pigmented lesions (5) including nevus (4) and malignant melanoma (1)	Compared with histologic findings, IC had a high positive predictive value (PPV) of 97.4% and a fair negative predictive value (NPV) of 52.9%, making it a good screening tool but inadequate gold standard. Moreover, IC is less sensitive for keratotic lesions and invasive disease.

de Nadai Barros et al. [18]	Brazil	2009	Transverse, observational	Pterygium (1) Actinic keratosis (AK) (9) Corneal/conjunctival intraepithelial neoplasia (CCIN) (9) SCC (20)	Cytological features related to malignancy were applied to determine an index score that best differentiates invasive SCC from preinvasive lesions. With an index score of ≥4.25, sensitivity was 95%, specificity was 93%, PPV was 95%, and NPV was 93% for predicting SCC. However, in two preinvasive lesions (one AK lesion and one CCIN lesion), IC sampling was not sufficiently deep to reach atypical cells and presents a limitation to diagnose disease affecting deeper tissue.

de Nadai Barros et al. [19]	Brazil	2014	Transverse, prospective, observational	Pterygia without atypical cells (19) Pterygia with associated OSSN (13)	IC showed high agreement with histopathology in detecting unsuspected OSSN in pterygia patients (sensitivity 92%, specificity 94%, PPV 92%, and NPV 94%).

**Table:** (b)

Vital dye staining					
Study	Location	Year	Study design	Number of eyes	Major findings
Romero et al. [20]	Brazil	2013	Prospective case series	Pterygia (10) Actinic keratosis (10) Conjunctival intraepithelial neoplasia and conjunctival SCC (27)	Toluidine blue 1% is a good tool for the diagnosis of OSSN and premalignant lesions but the intensity of staining does not correlate with the degree of malignancy (sensitivity 100%, specificity 50%, PPV 73%, and NPV 100%).

Steffen et al. [21]	South Africa	2014	Prospective diagnostic validation	Malignant lesions including invasive SCC (16) and severe dysplasia (17) Benign or premalignant lesions (42)	Methylene blue 1% can exclude malignant lesions but cannot replace histopathology as the gold standard (sensitivity 97%, specificity 50%, PPV 60%, and NPV 95%).

Gichuhi et al. [22]	Kenya	2015	Cross-sectional, multicenter	OSSN (143) Non-OSSN (276)	Toluidine blue 0.05% is a good screening tool but not a good diagnostic tool due to a high frequency of false positives (sensitivity 92%, specificity 31%, PPV 41%, and NPV 88%).

**Table:** (c)

Ultrasound biomicroscopy					
Study	Location	Year	Study design	Number of eyes	Major findings
Char et al. [23]	United States	2002	Prospective case series	SCC (4)	20 MHz high frequency ultrasound demonstrates deep involvement of tumor into the sclera, globe, or orbit and is a useful tool for tumors with deep invasion. It is of otherwise limited utility in tumors without deep extension.

Finger et al. [11]	United States	2003	Retrospective case series	Conjunctival intraepithelial neoplasia and SCC (11)	20 and/or 50 MHz high frequency ultrasound helps delineate tumor thickness, shape, and internal reflectivity and is particularly helpful in determining if the tumor has extended into the sclera, eye, and orbit.

**Table:** (d)

*In vivo* confocal microscopy (IVCM)					
Study	Location	Year	Study design	Number of eyes	Major findings
Parrozzani et al. [24]	Italy	2011	Prospective case series	Conjunctival intraepithelial neoplasia (2) Corneoconjunctival intraepithelial neoplasia (8)	IVCM findings of OSSN lesions were described. Structural findings included large areas of superficial cell debris and/or keratin debris accompanied by syncytial-like groupings, loss of the normal structure of the conjunctival epithelium and/or the corneal basal epithelium layer, papillomatous organization, large fibrovascular structures, and fine vessels perpendicular to the tumor surface. Marginal findings included subepithelial (pre-Bowman) space involvement in 4 cases, irregular healthy tissue infiltration at the lateral edge of the lesion in 2 cases, and abrupt demarcation between neoplastic cells and normal epithelium in 8 cases. *In vivo* cytomorphologic findings included cellular anisocytosis, pleocytosis, and anisonucleosis, enlarged nuclei with high nuclear to cytoplasmic ratio, high reflective cytoplasm, and indistinct cytoplasmic borders.

Alomar et al. [12]	United Kingdom and Italy	2011	Observational case series	Corneal/conjunctival intraepithelial neoplasia (CCIN) (4) Control (4; 2 with limbal stem cell deficiency, 1 with suspicious limbal lesion, 1 with diffuse keratoconjunctival proliferation)	This study defined IVCM features of CCIN, which included (1) hyperreflective pleomorphic cells of varying shapes and sizes, (2) the edge of hyperreflective CCIN lesion contrasting with the darker and smaller normal cells, (3) “starry night sky” pattern of the basal layer produced by prominent nucleoli, and (4) absence of subbasal corneal nerves within areas involved by CCIN compared to nonaffected regions. These IVCM findings were found to be highly correlated with histologic features.

Xu et al. [25]	China	2012	Retrospective case series	Conjunctival intraepithelial neoplasia (3) Carcinoma *in situ* (1) Ocular surface squamous carcinoma (1)	IVCM demonstrated cellular anisocytosis and enlarged nuclei with high nuclear to cytoplasmic ratio in conjunctival intraepithelial neoplasia while nests were partially formed by isolated keratinized, binucleated, and actively mitotic dysmorphic epithelial cells in carcinoma *in situ* and ocular surface squamous carcinoma. The IVCM characteristics were similar to histopathologic findings.

Nguena et al. [10]	Tanzania	2014	Case-control	OSSN (26) Benign conjunctival lesions (18) Age matched controls with normal conjunctiva (57)	IVCM was able to reliably distinguish normal conjunctiva from conjunctival lesions. However, IVCM was unable to reliably differentiate OSSN from benign conjunctival lesions due to an overlap in IVCM features between the two conditions (kappa = 0.44, 95% CI 0.32–0.57). IVCM has a low sensitivity (38.5%) and moderate specificity (66.7%) for distinguishing OSSN from benign conjunctival lesions.

**Table:** (e)

Anterior segment optical coherence tomography (AS-OCT)					
Study	Location	Year	Study design	Number of eyes	Major findings
Shousha et al. [13]	United States	2011	Prospective case series	Corneal/conjunctival intraepithelial neoplasia (CCIN) (7) Pterygia (7)	Ultra-high resolution (UHR) OCT demonstrated a thickened hyperreflective epithelium and abrupt transition from normal to hyperreflective epithelium in all CCIN cases. After medical treatment and clinical resolution, 4 cases demonstrated normal epithelial configuration on UHR-OCT while 3 cases showed residual disease that was clinically invisible. Continuation of treatment resulted in complete resolution of residual lesions on UHR-OCT.

Kieval et al. [26]	United States	2012	Prospective case series	Ocular surface squamous neoplasia (17) Pterygia (17)	UHR-OCT findings of OSSN and pterygia were well correlated to histopathologic findings. Both UHR-OCT and histopathologic specimens of OSSN demonstrated a thickened layer of epithelium, often with an abrupt transition from normal to neoplastic tissue. Meanwhile, both diagnostic modalities for pterygia showed a normal thin epithelium with underlying thickening of the subepithelial mucosal layers. Mean epithelial thickness on UHR-OCT for OSSN (346 *μ*m) was significantly higher than for pterygia (101 *μ*m) (*p* < 0.001). UHR-OCT had a high sensitivity (94%) and specificity (100%) for differentiating OSSN from pterygia.

Shousha et al. [27]	United States	2013	Prospective case series	OSSN (19) Primary acquired melanosis (8) Amelanotic melanoma (5) Nevi (2) Histiocytosis (1) Conjunctival lymphoma (6) Conjunctival amyloidosis (2) Pterygia (11)	UHR-OCT and histopathologic findings were closely correlated for all the examined ocular lesions. Specifically for OSSN, the epithelial layer was severely thickened and hyperreflective with an abrupt transition between normal and affected epithelium. In large lesions, a shadow from the hyperreflective epithelium may obscure the inferior border. The subepithelial layer was uninvolved in OSSN.

Nanji et al. [28]	United States	2015	Prospective case series	OSSN (21) Pterygia or pinguecula (24) Lymphoma (3) Pigmented conjunctival lesions (18) Salzmann nodular degeneration (6) Normal (10)	Commercially available HR-OCT is also capable of differentiating various lesions based on optical signs. Specifically in OSSN, HR-OCT shows epithelial thickening and hyperreflectivity.

**Table 2:** Summary of advantages and disadvantages of adjunctive diagnostic modalities in OSSN.

Imaging modality	Advantages	Disadvantages
Impression cytology	(1) Inexpensive (2) Easy to perform (3) Good correlation with histopathology	(1) Assesses only superficial cells and is unable to sample deep lesions or invasive disease (2) Requires skilled professional to interpret results

Vital dye staining	(1) Inexpensive (2) Easy to use (3) High sensitivity compared to histopathology making it a good screening tool	(1) Low to moderate specificity so a large number of benign lesions would also test positive

Ultrasound biomicroscopy	(1) Good width and depth of penetration allowing the detection of invasive disease and metastasis (2) Can be used for pigmented and thick lesions	(1) Lower resolution images compared to OCT (2) Requires skilled technician or provider to perform imaging (3) Need for eyebath and reclined position (4) Limited utility in noninvasive disease

*In vivo* confocal microscopy (IVCM)	(1) Allows microscopic and cellular examination of lesion (2) Images are en face	(1) Requires skilled technician or provider to perform test and interpret results (2) Unable to obtain cross-sectional images and thus may miss deep disease (3) Cannot obtain comprehensive scan of entire ocular surface (4) Difficult to obtain IVCM and pathology specimens from the same site (5) Overlap in features with benign ocular surface lesions limiting its use in differentiating OSSN from benign lesions

High resolution anterior segment optical coherence tomography	(1) High resolution images (2) Easy to use, noncontact (3) High specificity and sensitivity for differentiating OSSN from pterygia (4) HR-OCT morphologic features of OSSN are well defined, allowing the differentiation of OSSN from benign and other malignant ocular lesions (5) Ability to image the same site as before and therefore can be used to monitor disease resolution after treatment	(1) Limitation in width and depth of penetration, especially in commercial models (2) Shadowing in pigmented lesions and thick lesions, therefore limiting the ability to determine the posterior limit of lesions

## References

[1] Grossniklaus H. E., Green W. R., Luckenbach M., Chan C. C. (1987). Conjunctival lesions in adults. A clinical and histopathologic review. *Cornea*.

[2] Lee G. A., Hirst L. W. (1995). Ocular surface squamous neoplasia. *Survey of Ophthalmology*.

[3] Spraul C. W., Grossniklaus H. E. (1996). Tumors of the cornea and conjunctiva. *Current Opinion in Ophthalmology*.

[4] Erie J. C., Campbell R. J., Liesegang T. J. (1986). Conjunctival and corneal intraepithelial and invasive neoplasia. *Ophthalmology*.

[5] Hamam R., Bhat P., Foster C. S. (2009). Conjunctival/corneal intraepithelial neoplasia. *International Ophthalmology Clinics*.

[6] Yousef Y. A., Finger P. T. (2012). Squamous carcinoma and dysplasia of the conjunctiva and cornea: an analysis of 101 cases. *Ophthalmology*.

[7] Gichuhi S., Sagoo M. S., Weiss H. A., Burton M. J. (2013). Epidemiology of ocular surface squamous neoplasia in Africa. *Tropical Medicine and International Health*.

[8] Carreira H., Coutinho F., Carrilho C., Lunet N. (2013). HIV and HPV infections and ocular surface squamous neoplasia: systematic review and meta-analysis. *British Journal of Cancer*.

[9] Pizzarello L. D., Jakobiec F. A., Jakobiec F. A. (1978). Bowen's disease of the conjunctiva: a misnomer. *Ocular and Adnexal Tumors*.

[10] Nguena M. B., Van Den Tweel J. G., Makupa W. (2014). Diagnosing ocular surface squamous neoplasia in east africa: case-control study of clinical and in vivo confocal microscopy assessment. *Ophthalmology*.

[11] Finger P. T., Tran H. V., Turbin R. E. (2003). High-frequency ultrasonographic evaluation of conjunctival intraepithelial neoplasia and squamous cell carcinoma. *Archives of Ophthalmology*.

[12] Alomar T. S., Nubile M., Lowe J., Dua H. S. (2011). Corneal intraepithelial neoplasia: in vivo confocal microscopic study with histopathologic correlation. *American Journal of Ophthalmology*.

[13] Shousha M. A., Karp C. L., Perez V. L. (2011). Diagnosis and management of conjunctival and corneal intraepithelial neoplasia using ultra high-resolution optical coherence tomography. *Ophthalmology*.

[14] Nolan G. R., Hirst L. W., Wright R. G., Bancroft B. J. (1994). Application of impression cytology to the diagnosis of conjunctival neoplasms. *Diagnostic Cytopathology*.

[15] Tole D. M., McKelvie P. A., Daniell M. (2001). Reliability of impression cytology for the diagnosis of ocular surface squamous neoplasia employing the Biopore membrane. *British Journal of Ophthalmology*.

[16] Nolan G. R., Hirst L. W., Bancroft B. J. (2001). The cytomorphology of ocular surface squamous neoplasia by using impression. *Cancer*.

[17] Tananuvat N., Lertprasertsuk N., Mahanupap P., Noppanakeepong P. (2008). Role of impression cytology in diagnosis of ocular surface neoplasia. *Cornea*.

[18] de Nadai Barros J., Lowen M. S., Ballalai P. L., Mascaro V. L. D. M., Martins M. C. (2009). Predictive index to differentiate invasive squamous cell carcinoma from preinvasive ocular surface lesions by impression cytology. *British Journal of Ophthalmology*.

[19] de Nadai Barros J., Lowen M. S., de Moraes-Filho M. N., Martins M. C. (2014). Use of impression cytology for the detection of unsuspected ocular surface squamous neoplasia cells in pterygia. *Arquivos Brasileiros de Oftalmologia*.

[20] Romero I. L., Barros J. D. N., Martins M. C., Ballalai P. L. (2013). The use of 1% toluidine blue eye drops in the diagnosis of ocular surface squamous neoplasia. *Cornea*.

[21] Steffen J., Rice J., Lecuona K., Carrara H. (2014). Identification of ocular surface squamous neoplasia by in vivo staining with methylene blue. *British Journal of Ophthalmology*.

[22] Gichuhi S., Macharia E., Kabiru J. (2015). Toluidine blue 0.05% vital staining for the diagnosis of ocular surface squamous neoplasia in Kenya. *JAMA Ophthalmology*.

[23] Char D. H., Kundert G., Bove R., Crawford J. B. (2002). 20 MHz high frequency ultrasound assessment of scleral and intraocular conjunctival squamous cell carcinoma. *British Journal of Ophthalmology*.

[24] Parrozzani R., Lazzarini D., Dario A., Midena E. (2011). In vivo confocal microscopy of ocular surface squamous neoplasia. *Eye*.

[25] Xu Y., Zhou Z., Xu Y. (2012). The clinical value of in vivo confocal microscopy for diagnosis of ocular surface squamous neoplasia. *Eye*.

[26] Kieval J. Z., Karp C. L., Shousha M. A. (2012). Ultra-high resolution optical coherence tomography for differentiation of ocular surface squamous neoplasia and pterygia. *Ophthalmology*.

[27] Shousha M. A., Karp C. L., Canto A. P. (2013). Diagnosis of ocular surface lesions using ultra-high-resolution optical coherence tomography. *Ophthalmology*.

[28] Nanji A. A., Sayyad F. E., Galor A., Dubovy S., Karp C. L. (2015). High-resolution optical coherence tomography as an adjunctive tool in the diagnosis of corneal and conjunctival pathology. *The Ocular Surface*.

[29] de Nadai Barros J., Araújo de Almeida S. R., Lowen M. S., da Cunha M. C., Gomes J. Á. P. (2015). Impression cytology in the evaluation of ocular surface tumors: review article. *Arquivos Brasileiros de Oftalmologia*.

[30] Pavlin C. J., Sherar M. D., Foster F. S. (1990). Subsurface ultrasound microscopic imaging of the intact eye. *Ophthalmology*.

[31] Bianciotto C., Shields C. L., Guzman J. M. (2011). Assessment of anterior segment tumors with ultrasound biomicroscopy versus anterior segment optical coherence tomography in 200 cases. *Ophthalmology*.

[32] Pavlin C. J., Harasiewicz K., Sherar M. D., Foster F. S. (1991). Clinical use of ultrasound biomicroscopy. *Ophthalmology*.

[33] Pavlin C. J. (1995). Practical application of ultrasound biomicroscopy. *Canadian Journal of Ophthalmology*.

[34] Chiou A. G.-Y., Kaufman S. C., Kaufman H. E., Beuerman R. W. (2006). Clinical corneal confocal microscopy. *Survey of Ophthalmology*.

[35] Duchateau N., Hugol D., D'Hermies F. (2005). Contribution of in vivo confocal microscopy to limbal tumor evaluation. *Journal Francais d'Ophtalmologie*.

[36] Malandrini A., Martone G., Traversi C., Caporossi A. (2008). In vivo confocal microscopy in a patient with recurrent conjunctival intraepithelial neoplasia. *Acta Ophthalmologica*.

[37] Wakuta M., Chikama T.-I., Takahashi N., Nishida T. (2008). A case of bilateral corneal epithelial dysplasia characterized by laser confocal biomicroscopy and cytokeratin immunofluorescence. *Cornea*.

[38] Falke K., Zhivov A., Zimpfer A., Stachs O., Guthoff R. F. (2012). Diagnosis of conjunctival neoplastic lesions by confocal in-vivo microscopy. *Klinische Monatsblätter für Augenheilkunde*.

[39] Balestrazzi A., Martone G., Pichierri P., Tosi G. M., Caporossi A. (2008). Corneal invasion of ocular surface squamous neoplasia after clear corneal phacoemulsification: in vivo confocal microscopy analysis. *Journal of Cataract & Refractive Surgery*.

[40] Gentile C. M., Burchakchi A. I., Oscar C. J. (2009). In vivo confocal microscopy study of ocular surface neoplasia manifesting after radial keratotomy and laser in situ keratomileusis. *Cornea*.

[41] Izatt J. A., Hee M. R., Swanson E. A. (1994). Micrometer-scale resolution imaging of the anterior eye in vivo with optical coherence tomography. *Archives of Ophthalmology*.

[42] Thomas B. J., Galor A., Nanji A. A. (2014). Ultra high-resolution anterior segment optical coherence tomography in the diagnosis and management of ocular surface squamous neoplasia. *Ocular Surface*.

[43] Wang J., Abou Shousha M., Perez V. L. (2011). Ultra-high resolution optical coherence tomography for imaging the anterior segment of the eye. *Ophthalmic Surgery, Lasers and Imaging Retina*.

[44] Ramos J. L. B., Li Y., Huang D. (2009). Clinical and research applications of anterior segment optical coherence tomography—a review. *Clinical and Experimental Ophthalmology*.

[45] Vajzovic L. M., Karp C. L., Haft P. (2011). Ultra high-resolution anterior segment optical coherence tomography in the evaluation of anterior corneal dystrophies and degenerations. *Ophthalmology*.

